# Vaginal Infection in Pregnancy Is Associated with Amniotic Oxidative Stress: Insights from AOPP and MDA on Neonatal Outcomes

**DOI:** 10.3390/antiox15091123

**Published:** 2026-09-04

**Authors:** Meryem Kececi Oguzhanoglu, Icten Olgu Bafali, Kursat Oguzhanoglu, Senem Karacabey Cakmak, Busra Seker Atas, Muhammed Oguz Yildiz, Ali Cetin

**Affiliations:** 1 Department of Obstetrics and Gynecology, Haseki Training and Research Hospital, University of Health Sciences, 34265 Istanbul, Türkiyekursatoguzhanoglu@gmail.com (K.O.); senemkaracabey@gmail.com (S.K.C.); bussraseker@hotmail.com (B.S.A.); dralicetin@yahoo.com (A.C.); 2 Department of Biochemistry, Haseki Training and Research Hospital, University of Health Sciences, 34265 Istanbul, Türkiye; droguzyildiz@hotmail.com

**Keywords:** advanced oxidation protein products, malondialdehyde, vaginal infection, bacterial vaginosis, amniotic fluid, oxidative stress, neonatal outcome

## Abstract

Amniotic oxidative stress in women with vaginal infection but no documented intra-amniotic infection has received little attention. In this prospective cohort study of 90 women undergoing elective cesarean delivery, 45 had symptomatic, culture- or Nugent-confirmed vaginal infection and 45 were asymptomatic controls. Amniotic fluid advanced oxidation protein products (AOPP) and malondialdehyde (MDA) were measured by commercial ELISA. Both were higher in the infection group (median 7.96 versus 5.77 ng/mL and 12.49 versus 7.73 nmol/mL, both *p* < 0.001), with lower cord blood pH (*p* = 0.003) and more frequent neonatal intensive care unit (NICU) admission (26.7% versus 4.4%, *p* = 0.007). AOPP was associated with NICU admission (area under the curve 0.917, 95% CI 0.777 to 0.999), although this rests on 14 events with thresholds derived and evaluated in the same sample. Elevations were largest in the bacterial vaginosis and aerobic bacterial subgroups, but the etiologies did not differ. Both kits were designed for serum and are not validated for amniotic fluid, and 16.7% of MDA measurements fell outside the calibration range, so the MDA results are semi-quantitative. Vaginal infection at cesarean delivery is associated with higher amniotic oxidative stress markers; these findings are exploratory and require external validation.

## 1. Introduction

Vaginal infections, including bacterial vaginosis, candidiasis, trichomoniasis, and aerobic vaginitis, are among the most common gynecological conditions encountered during pregnancy. Ascending spread from the lower genital tract into the amniotic cavity has been proposed as a route by which such infections could provoke a local inflammatory and oxidative response, although this pathway was not assessed in the present study. Intra-amniotic infection and inflammation have been consistently linked to preterm birth, chorioamnionitis, and a spectrum of adverse neonatal outcomes, yet clinical diagnosis remains challenging because overt maternal symptoms are frequently absent or nonspecific [1,2].

The clinical scale of this problem is substantial, although estimates vary by population and diagnostic method. In the United States, reported prevalence of bacterial vaginosis among pregnant women ranges from 5.8% to 19.3% and is higher in some racial and ethnic groups [2]. Aerobic vaginitis, a distinct dysbiotic entity characterized by an overtly inflammatory rather than anaerobic-suppressive vaginal flora, has a reported prevalence of 4% to 8% in pregnancy [3]. Vulvovaginal candidiasis is even more common, affecting up to 20% of pregnant women and peaking near 30% in the third trimester [4]. Trichomonas vaginalis continues to represent a substantial global reproductive health burden, with an estimated 156 million new infections occurring worldwide each year among adults aged 15 to 49 years [5]. Taken together, these figures indicate that a considerable proportion of pregnancies are affected by at least one form of vaginal infection.

Oxidative stress, defined as an imbalance between reactive oxygen species production and antioxidant defense capacity, has been proposed as a mechanistic link between maternal infection and fetal compromise. Advanced oxidation protein products (AOPP) and malondialdehyde (MDA) are among the most widely used biomarkers of protein and lipid oxidative damage, respectively. Serum AOPP concentrations measured in the first trimester have been reported to be higher than in non-pregnant female controls [6], and related oxidative markers are elevated in pregnancy complications such as gestational diabetes mellitus, where they reflect disrupted redox homeostasis [7]. Within the amniotic compartment specifically, prior work using mass-spectrometry-based markers demonstrated that intra-amniotic infection, classified using clinical, microbiological and histological criteria, is accompanied by a measurable rise in amniotic fluid oxidative stress signatures compared with uninfected pregnancies [8].

Three gaps remain. First, previous amniotic fluid studies have examined histologically or clinically confirmed intra-amniotic infection, whereas much less is known about the more common clinical situation of maternal vaginal infection without confirmed intra-amniotic spread. Second, vaginal infections are etiologically heterogeneous, comprising bacterial, fungal and protozoal entities that provoke distinct local immune and inflammatory responses; whether amniotic oxidative stress is elevated uniformly across these etiologies, or disproportionately in some, has not to our knowledge been examined. Third, AOPP and MDA are widely used and inexpensive to measure, but they have not been evaluated together in amniotic fluid in relation to neonatal outcomes such as neonatal intensive care unit (NICU) admission in this setting.

This prospective cohort study had three aims. The first was to determine whether vaginal infection is associated with higher amniotic fluid AOPP and MDA concentrations in women undergoing cesarean delivery. The second was to examine the association between these biomarkers and neonatal outcomes, specifically umbilical cord blood pH and NICU admission. The third was to explore whether any such association differs across vaginal infection etiologies.

We hypothesized that vaginal infection would be accompanied by higher amniotic AOPP and MDA concentrations and by less favorable neonatal outcomes, and that the difference would be most pronounced in etiologies characterized by a strong local neutrophilic or dysbiotic response. The study was designed to describe associations rather than to establish a causal pathway or to develop a clinical prediction tool.

## 2. Materials and Methods

### 2.1. Study Design and Setting

This prospective cohort study was conducted in the Department of Obstetrics and Gynecology at the University of Health Sciences, Haseki Training and Research Hospital, Istanbul, Türkiye, a single tertiary-care center. The study protocol was approved by the Clinical Research Ethics Committee of Haseki Training and Research Hospital (approval number 226-2022, approval date 25 January 2023) and was conducted in accordance with the Declaration of Helsinki. Written informed consent was obtained from all participants. Reporting followed the Strengthening the Reporting of Observational Studies in Epidemiology (STROBE) guidelines for cohort studies [9].

### 2.2. Participants

Women scheduled for cesarean delivery who underwent routine pre-operative vaginal examination were screened for eligibility between February and May 2023. Eligible participants were 18–45 years of age with a singleton pregnancy and not in active labor, and had received no antimicrobial treatment for vaginal infection before the vaginal swab was obtained. Women with diabetes mellitus, hypertensive disorders of pregnancy, multiple pregnancy, other comorbidities, or a history of smoking were excluded, as these conditions are independently associated with increased oxidative stress and would confound biomarker interpretation. Of 331 women assessed, 53 were excluded before sampling: 23 for smoking, 16 for gestational diabetes mellitus, 9 for gestational hypertension, 3 for twin pregnancy and 2 for type 2 diabetes mellitus. No vaginal or amniotic fluid samples were obtained from excluded women.

Vaginal swabs were obtained during the routine pre-operative examination, 1 to 3 h before cesarean delivery, and were transported immediately to the hospital microbiology laboratory. All specimens underwent routine culture and Gram staining as part of standard clinical care; laboratory staff were unaware of study participation. Bacterial vaginosis was diagnosed by Nugent scoring of Gram-stained smears (score 7 to 10) [10]. Trichomonas vaginalis was detected by culture in Diamond’s medium. Yeast isolates were identified to species level using the VITEK 2 system (bioMérieux, Marcy-l’Étoile, France); because culture positivity alone does not distinguish infection from colonisation, candidiasis required a positive yeast culture together with compatible vaginal symptoms. Cases yielding aerobic bacterial pathogens were classified on the basis of symptoms together with predominant or pure growth on culture. Routine Gram-stain reports did not include leucocyte counts, lactobacillary grade or the proportion of parabasal cells, so the Donders aerobic vaginitis score [11] could not be calculated. This subgroup is therefore designated symptomatic aerobic bacterial growth and is treated as a descriptive category rather than as a defined diagnosis of aerobic vaginitis.

Women presenting with vaginal symptoms (discharge, itching, burning) who additionally had a Nugent score of 7 to 10 and/or a positive vaginal culture were allocated to the infection group (Group 1). Asymptomatic women without clinical leukorrhea, with a negative vaginal culture and a Nugent score of 0 to 3 indicating normal vaginal flora, were allocated to the control group (Group 2). Women with intermediate Nugent scores, asymptomatic dysbiosis or asymptomatic candidal colonisation met the criteria for neither group and were not enrolled. The comparison therefore contrasts the two ends of a continuum rather than adjacent categories, and infection status is entangled with symptomatic status by design.

Culture results became available approximately 3 days after sampling. Neither the obstetric nor the neonatal team had access to microbiological results at the time of cesarean section or when the decision to admit to the neonatal intensive care unit was made. Because results entered the clinical record thereafter, they may have informed subsequent management in neonates with prolonged admissions.

Recruitment and group allocation proceeded concurrently. Amniotic fluid was collected from all 278 women screened during the study period, of whom 45 met the criteria for the infection group and 233 were not infected. The control group reached its pre-specified size of 45 first and was then closed; recruitment continued until the infection group also reached 45, at which point enrolment ended. Samples from the remaining 188 uninfected women were discarded without analysis. Written informed consent was obtained from all 278 women before sampling.

All cesarean deliveries were elective. The indication was a previous cesarean section in 87 women (96.7%) and breech presentation in 3 (3.3%); no delivery was performed for fetal distress or another emergency indication, and the distribution of indications did not differ between groups (*p* = 1.00). No woman was in labor, membranes were intact in all cases, and amniotomy was performed at surgery, so the duration of membrane rupture was effectively zero for every participant. All women received a single 2 g dose of intravenous cephalosporin as standard surgical prophylaxis before skin incision in accordance with unit protocol; this was uniform across both groups. Group B streptococcus screening is not performed routinely at our institution and was therefore not available.

A complete blood count was obtained preoperatively in all participants as part of routine care. The white blood cell count did not differ between groups, and no participant had a count above 15 × 10^9^/L. No participant had maternal fever or other systemic signs of infection, and clinical chorioamnionitis was not suspected in any case. C-reactive protein is not measured routinely in this setting and was therefore not available.

### 2.3. Sample Size

The sample size was calculated a priori using G*Power software (v3.1) [12]. Assuming an effect size of 0.6, α = 0.05 and power (1−β) = 0.80 for a two-tailed independent-samples comparison of mean biomarker concentrations between the infection and control groups, a total of 90 participants (45 per group) was required. The effect size of 0.6 was not derived from pilot data or from a specific published estimate; because no prior study had reported amniotic fluid AOPP or MDA concentrations in women with vaginal infection, it was adopted as a conventional medium-to-large effect considered clinically meaningful. AOPP and MDA were treated jointly as co-primary outcomes. Because the observed distributions were non-normal, the primary analyses used non-parametric tests, which carry a modest loss of efficiency relative to the parametric test assumed in the calculation; the corresponding requirement would therefore be marginally higher than the figure obtained.

### 2.4. Specimen Collection and Processing

Following hysterotomy and rupture of the membranes at cesarean delivery, approximately 10 mL of amniotic fluid was aspirated under sterile conditions. Samples were inspected visually at the time of collection; no sample showed macroscopic blood contamination, and any sample with visible contamination would have been excluded. Samples were processed without delay in a research centrifuge located within the delivery theatre (Eppendorf 5415C; Eppendorf AG, Hamburg, Germany) at 3000 rpm for 20 min at ambient temperature. The supernatant was aliquoted and stored at −80 °C in the unit’s dedicated research freezer until batch analysis. The total interval between collection and freezing, including centrifugation, did not exceed 30 min. No antioxidant preservative was added. Storage duration ranged from 1 week to 4 months; no sample underwent a freeze–thaw cycle, and all samples were assayed in a single batch.

### 2.5. Biochemical Analysis

Amniotic fluid concentrations of AOPP and MDA were measured at an independent external laboratory (Opakgen Research Laboratory, Istanbul, Türkiye) using commercial ELISA kits (Bioassay Technology Laboratory, Shanghai, China; Human AOPP, Cat. No. E1266Hu; Human MDA, Cat. No. E1371Hu), following the manufacturer’s instructions. Assay selection and the analytical protocol were determined by the study biochemist. Samples were submitted under randomly assigned identification numbers and laboratory personnel had no access to clinical or group information, so the analyst was necessarily blinded to allocation.

The manufacturer describes both assays as biotin double-antibody sandwich enzyme immunoassays with absorbance read at 450 nm, in which optical density increases with analyte concentration. According to technical information supplied with the kits, the AOPP assay uses a matched antibody pair: a monoclonal capture antibody and a biotinylated polyclonal detection antibody. These are directed against chlorinated and aggregated protein epitopes formed when hypochlorous acid modifies serum proteins, principally albumin, and are stated not to bind native, non-oxidized serum proteins. It is calibrated against purified human AOPP oxidized in vitro under controlled conditions and supplied in lyophilised form. The MDA assay is described as using a matched antibody pair of the same configuration, directed against malondialdehyde generated by radical-mediated breakdown of polyunsaturated fatty acids, calibrated against a lyophilised human MDA standard. These descriptions were provided by the manufacturer and could not be verified independently. One element of them is difficult to reconcile with the analyte: a sandwich format requires two antibodies to bind simultaneously to distinct epitopes on the same molecule, which is not feasible for malondialdehyde, a three-carbon dialdehyde of approximately 72 Da. We report the manufacturer’s description as given, but note that the stated format is not consistent with the size of this analyte.

The stated analytical ranges were 1.5 to 32 ng/mL for AOPP and 2.5 to 40 nmol/mL for MDA, with limits of detection of 0.1 ng/mL and 0.14 nmol/mL respectively. Manufacturer-reported intra-assay and inter-assay coefficients of variation were below 8% and below 10% for both kits; samples were assayed in single wells, so study-specific coefficients of variation could not be calculated. Samples were assayed undiluted. Kit lot numbers were not recorded in the study documentation at the time of analysis and could not be retrieved retrospectively.

Both kits are labelled for research use only and are not intended for clinical diagnosis. The manufacturer lists serum, plasma, cell culture supernatant, cell lysate, tissue homogenate and other biological fluids as acceptable sample types, without specifying amniotic fluid. Neither has been validated for amniotic fluid, and no dilutional linearity or spike-recovery testing was performed in this matrix. The calibration basis of the AOPP assay, purified protein oxidized in vitro, differs from the chloramine-T equivalence reported by the established Witko-Sarsat spectrophotometric method [13]. Values obtained by the two methods are therefore not interchangeable, and absolute concentrations should not be compared with values reported for other assays or matrices. All comparisons in this study are within-study and method-specific. The proportion of results falling outside the calibration range is reported in Section 3.

### 2.6. Outcome Variables

Maternal demographic and clinical variables (age, gravidity, parity, body mass index, education level, pre-pregnancy contraceptive method, indication for cesarean section) and perinatal outcomes (gestational age at delivery, birth weight, 1 min and 5 min Apgar scores, umbilical cord blood pH, and NICU admission) were recorded for all participants. For neonates admitted to the NICU, the admission diagnosis and length of stay were also recorded.

The decision to admit a neonate to the intensive care unit was made solely by the attending neonatal team, independently of the study. The unit applies standard indications consistent with international practice: prematurity or low birth weight, respiratory distress, suspected sepsis or serious infection, hypoglycaemia and other metabolic disturbances, perinatal asphyxia or neurological signs, cardiac or circulatory problems, surgical and gastrointestinal conditions, and difficulties with feeding or thermoregulation. The research team had no role in admission decisions, subsequent management or timing of discharge; admission status, diagnosis and length of stay were recorded observationally from the clinical record.

### 2.7. Statistical Analysis

Statistical analyses were performed using Python (v3.11; SciPy v1.13, statsmodels v0.14, scikit-learn v1.5). The normality of continuous variables was assessed using the Shapiro–Wilk test. Normally distributed variables were compared between groups using the independent-samples *t*-test and are presented as mean ± standard deviation, with Cohen’s d as the effect size. Non-normally distributed variables were compared using the Mann–Whitney U test and are presented as median (interquartile range [IQR]), with the rank-biserial correlation (r) as the effect size [14]. Throughout, r is signed so that a positive value indicates a higher value in the infection group and a negative value a higher value in controls. Categorical variables were compared using the chi-square or Fisher’s exact test, as appropriate. Spearman correlation assessed associations between biomarkers and continuous perinatal outcomes.

Receiver operating characteristic (ROC) curve analysis was used to describe the discriminative performance of AOPP and MDA for vaginal infection status and for NICU admission. Thresholds were identified by the Youden index. Thresholds were both derived and evaluated in the same sample. Confidence intervals (95%) for the area under the curve, and the 2.5th to 97.5th percentile range of each threshold, were therefore obtained from 4000 bootstrap resamples. Optimism-corrected estimates of the area under the curve, sensitivity and specificity were calculated by the same procedure. Sensitivity, specificity, likelihood ratios and, for NICU admission only, predictive values are reported with 95% confidence intervals; predictive values are not reported for infection status because the 1:1 design fixes prevalence. The areas under the curve for AOPP and MDA were compared using the DeLong test for correlated curves.

Differences in biomarker levels across the six groups (five infection etiologies and controls) were assessed using the Kruskal–Wallis test, followed by Dunn’s post hoc test [15]. Bonferroni correction was applied to all fifteen pairwise comparisons among the six groups; the five comparisons against controls are reported together with uncorrected *p*-values. A supplementary Kruskal–Wallis test restricted to the five infection etiologies, excluding controls, was performed to test whether the etiological subgroups differed from one another. The minimum difference detectable at 80% power was calculated for each subgroup. A sensitivity analysis restricted to the isolated bacterial vaginosis subgroup versus controls was performed to assess the robustness of the main finding in the most etiologically homogeneous subgroup. Two further sensitivity analyses addressed biomarker values falling outside the manufacturer’s calibration range: the first excluded them, and the second treated them as censored by assigning the nearer calibration boundary.

The multivariable model for NICU admission was fitted by Firth penalized logistic regression, which reduces small-sample bias when the number of events is limited; profile penalized-likelihood confidence intervals are reported. With 14 events, this analysis is exploratory rather than predictive, and no claim of clinical prediction is made. Vaginal infection status lies upstream of both biomarkers and the outcome rather than on the causal path between them, so it was additionally entered as a candidate covariate rather than excluded [16]. Given the exploratory nature of the correlation, ROC and regression analyses beyond the prespecified primary group comparisons, formal multiplicity correction was applied to the etiology-stratified comparisons only; other exploratory analyses should be interpreted with corresponding caution. A two-sided *p*-value <0.05 was considered statistically significant.

## 3. Results

### 3.1. Baseline Characteristics

**Figure 1:**
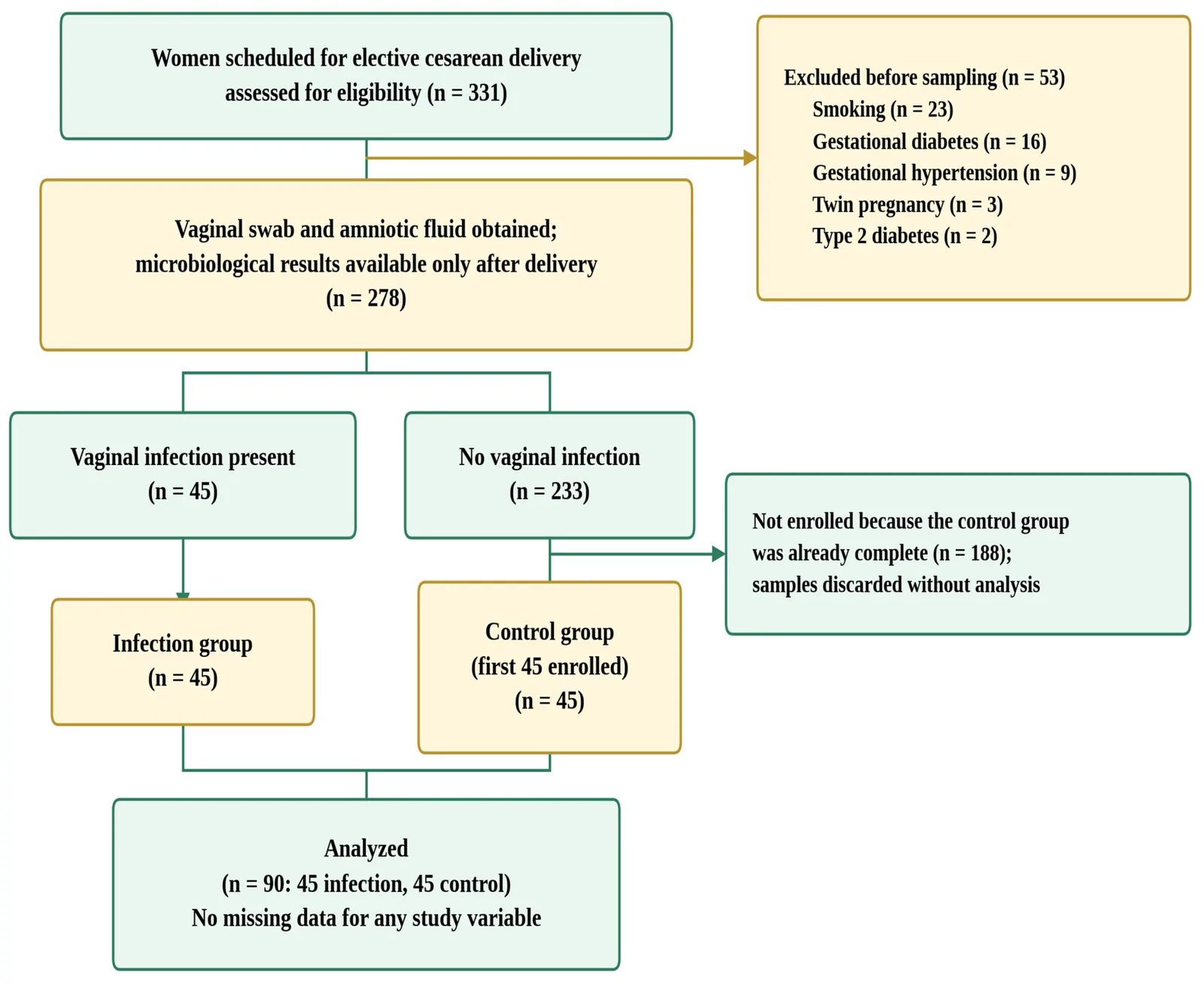
Flow of participants through the study. Numbers of women assessed for eligibility, excluded with reasons, sampled, allocated to groups and analyzed. Women excluded before sampling did not undergo vaginal or amniotic fluid sampling. No woman declined participation.

Of 331 women assessed for eligibility, 53 were excluded before sampling for the reasons given in the Methods, leaving 278 who had a vaginal swab and an amniotic fluid sample obtained. Of these, 45 met the criteria for the infection group and 233 were uninfected. The first 45 uninfected women formed the control group, after which that arm was closed; recruitment continued until the infection group also reached 45. Samples from the remaining 188 uninfected women were discarded without analysis, the sole reason for their exclusion being that the control group was already complete. No woman withdrew after sampling, and the 90 analyzed participants had no missing data for any study variable. Participant flow is shown in Figure 1.

Baseline demographic and clinical characteristics are summarized in Table 1. The two groups did not differ significantly in age, gravidity, parity or body mass index. Education level was comparable: primary school 21 (46.7%) versus 20 (44.4%), middle school 15 (33.3%) versus 15 (33.3%), high school 5 (11.1%) versus 7 (15.6%), and university or vocational higher education 4 (8.9%) versus 3 (6.7%) in the infection and control groups respectively (*p* = 0.731). Pre-pregnancy contraceptive method was also comparable: withdrawal (coitus interruptus) 24 (53.3%) versus 28 (62.2%), condom 13 (28.9%) versus 11 (24.4%), oral contraceptive 5 (11.1%) versus 2 (4.4%), intrauterine device 2 (4.4%) versus 4 (8.9%), and no method 1 (2.2%) versus 0 (*p* = 0.489).

All participants were free of diabetes, hypertensive disease and smoking by design, which removes these three sources of confounding; it does not eliminate confounding in general, and residual differences in unmeasured characteristics cannot be excluded. Baseline *p*-values above 0.05 indicate only that the study was unable to demonstrate a difference, and the three-year difference in median age (*p* = 0.125) illustrates this limitation.

### 3.2. Amniotic Fluid Oxidative Stress Markers

We next compared amniotic fluid oxidative stress between groups. AOPP and MDA concentrations were significantly higher in the infection group than in controls (Table 2), with large effect sizes (r = 0.56 and r = 0.62, respectively).

**Figure 2:**
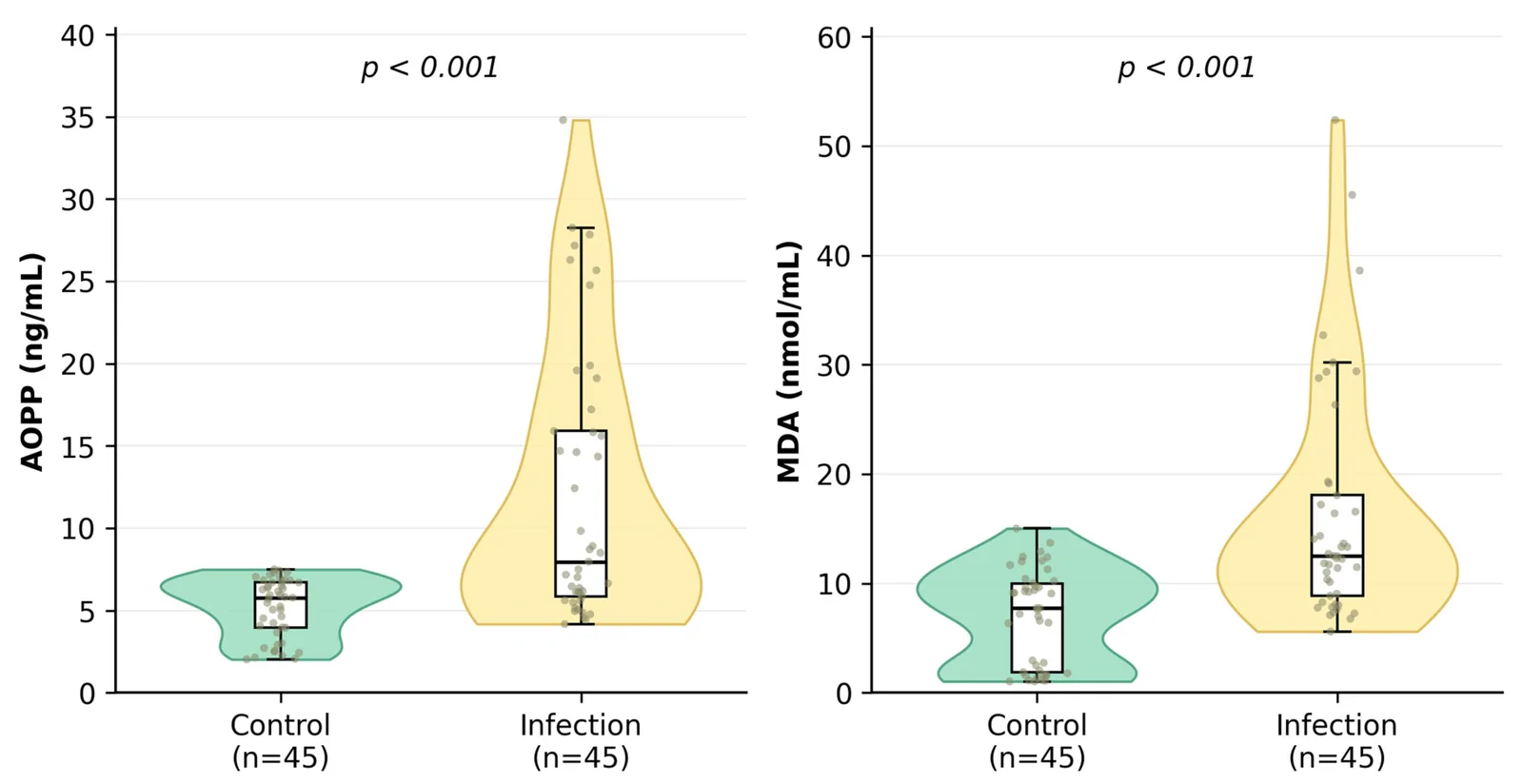
Amniotic fluid AOPP and MDA by vaginal infection status. Violin plots show the distribution of values in women with vaginal infection (*n* = 45) and controls (*n* = 45); the inner box plots indicate the median and interquartile range with whiskers extending to 1.5 times the interquartile range, and individual points represent single participants. Axes are not truncated. *p*-values are from the Mann–Whitney U test. In both panels, green denotes controls and gold denotes the infection group.

This difference is illustrated in Figure 2. AOPP and MDA were positively correlated with each other (Spearman ρ = 0.48, *p* < 0.001), consistent with the two biomarkers reflecting overlapping, though mechanistically distinct, dimensions of oxidative injury: protein oxidation and lipid peroxidation, respectively.

No result fell below the limit of detection for either assay: the lowest observed values were 2.02 ng/mL for AOPP (limit of detection 0.1 ng/mL) and 1.03 nmol/mL for MDA (limit of detection 0.14 nmol/mL). Results falling outside the manufacturer’s calibration range were examined separately. One AOPP value (1.1%) exceeded the upper limit of 32 ng/mL. For MDA, 13 values (14.4%) fell below the lower limit of 2.5 nmol/mL and 2 values (2.2%) exceeded the upper limit of 40 nmol/mL, giving 15 of 90 measurements (16.7%) outside the calibration range. All 13 sub-range MDA values occurred in the control group. Values below the lowest standard are extrapolated from the calibration curve rather than measured against it, so approximately one third of the control distribution is not quantified in the ordinary sense and the MDA results are best regarded as semi-quantitative. This pattern is compatible with a measuring range calibrated for serum, but it is equally compatible with degradation over longer storage, with analytical extrapolation, or with a true biological difference, and the present data cannot distinguish these. Two sensitivity analyses were therefore performed. Restricting the comparison to measurements within the calibration range attenuated the group difference in MDA (median 12.29 versus 9.32 nmol/mL, *p* = 0.001, r = 0.44), whereas AOPP was essentially unchanged (7.72 versus 5.77 ng/mL, *p* < 0.001, r = 0.55). This analysis runs against the study hypothesis, since removing low control values raises the control median and narrows the difference, but it discards 13 controls. In a second analysis, the out-of-range values were instead treated as censored and assigned the nearer calibration boundary, which retains all 90 participants; the group difference was then identical to that in the full dataset (median 12.49 versus 7.73 nmol/mL, *p* < 0.001, r = 0.62), because rank-based comparison is insensitive to the exact values assigned below the threshold. The MDA effect size is therefore bounded by r = 0.44 and r = 0.62.

### 3.3. Etiology-Specific Patterns

To describe whether this biomarker elevation was uniform across causative organisms, the infection group was stratified into five etiological subgroups and compared with controls (Table 3). Across the six groups, the Kruskal–Wallis test indicated a significant overall difference for both biomarkers (AOPP: H = 29.31, df = 5, *p* = 2.0 × 10^−5^; MDA: H = 29.44, df = 5, *p* = 1.9 × 10^−5^). After Bonferroni correction, bacterial vaginosis and symptomatic aerobic bacterial growth remained significantly elevated relative to controls for both biomarkers, whereas the mixed, trichomoniasis and candidiasis subgroups did not.

Two qualifications apply to this pattern. First, a supplementary Kruskal–Wallis test restricted to the five infection etiologies, excluding controls, did not demonstrate a difference between the subgroups themselves (AOPP: H = 9.19, df = 4, *p* = 0.056; MDA: H = 5.61, df = 4, *p* = 0.230). The data therefore show that individual subgroups differ from controls to varying degrees, but do not establish that the etiologies differ from one another. Second, the subgroups were small and correspondingly underpowered: at 80% power, the minimum detectable difference ranged from 6.8 to 9.5 ng/mL for AOPP and from 9.2 to 12.9 nmol/mL for MDA, corresponding to standardized effect sizes of approximately 1.0. Median MDA was numerically higher than in controls in both the trichomoniasis and candidiasis subgroups (11.40 and 10.35 versus 7.73 nmol/mL; uncorrected *p* = 0.046 and 0.023), so these are findings that could not be demonstrated rather than differences shown to be absent.

A sensitivity analysis restricted to the isolated bacterial vaginosis subgroup (*n* = 10) versus controls (*n* = 45) was consistent with this pattern (AOPP: *p* = 0.0012, r = 0.66; MDA: *p* = 0.0003, r = 0.75).

### 3.4. Perinatal Outcomes and Biomarker Discrimination

Perinatal outcomes are presented in Table 4. Umbilical cord blood pH was significantly lower and NICU admission significantly more frequent in the infection group, whereas gestational age at delivery and birth weight did not differ. Apgar scores were nominally lower in the infection group at 1 and 5 min (*p* = 0.044 and *p* = 0.045). The medians were, however, identical or nearly identical between groups (9 versus 9 and 10 versus 10), and the comparisons were not corrected for multiplicity. These differences are too fragile to be treated as evidence of adverse neonatal outcome.

Fourteen neonates were admitted to the NICU, 12 in the infection group and 2 in the control group. Nine admissions were for transient tachypnoea of the newborn (7 infection, 2 control), with a median stay of 7 days (range 3 to 12), and five were for clinical sepsis with negative cultures (all in the infection group), with a median stay of 14 days (range 10 to 20). No admission was for prematurity, congenital anomaly or surgical indication. The median stay of seven days for transient tachypnoea is longer than the 24 to 72 h over which the condition typically resolves, and reflects local unit practice on observation and discharge rather than the duration of respiratory symptoms.

Both AOPP and MDA showed a weak inverse correlation with cord blood pH (AOPP: ρ = −0.24, *p* = 0.023; MDA: ρ = −0.24, *p* = 0.021), corresponding to approximately 6% of shared variance; neither biomarker correlated significantly with birth weight or gestational age at delivery.

**Figure 3:**
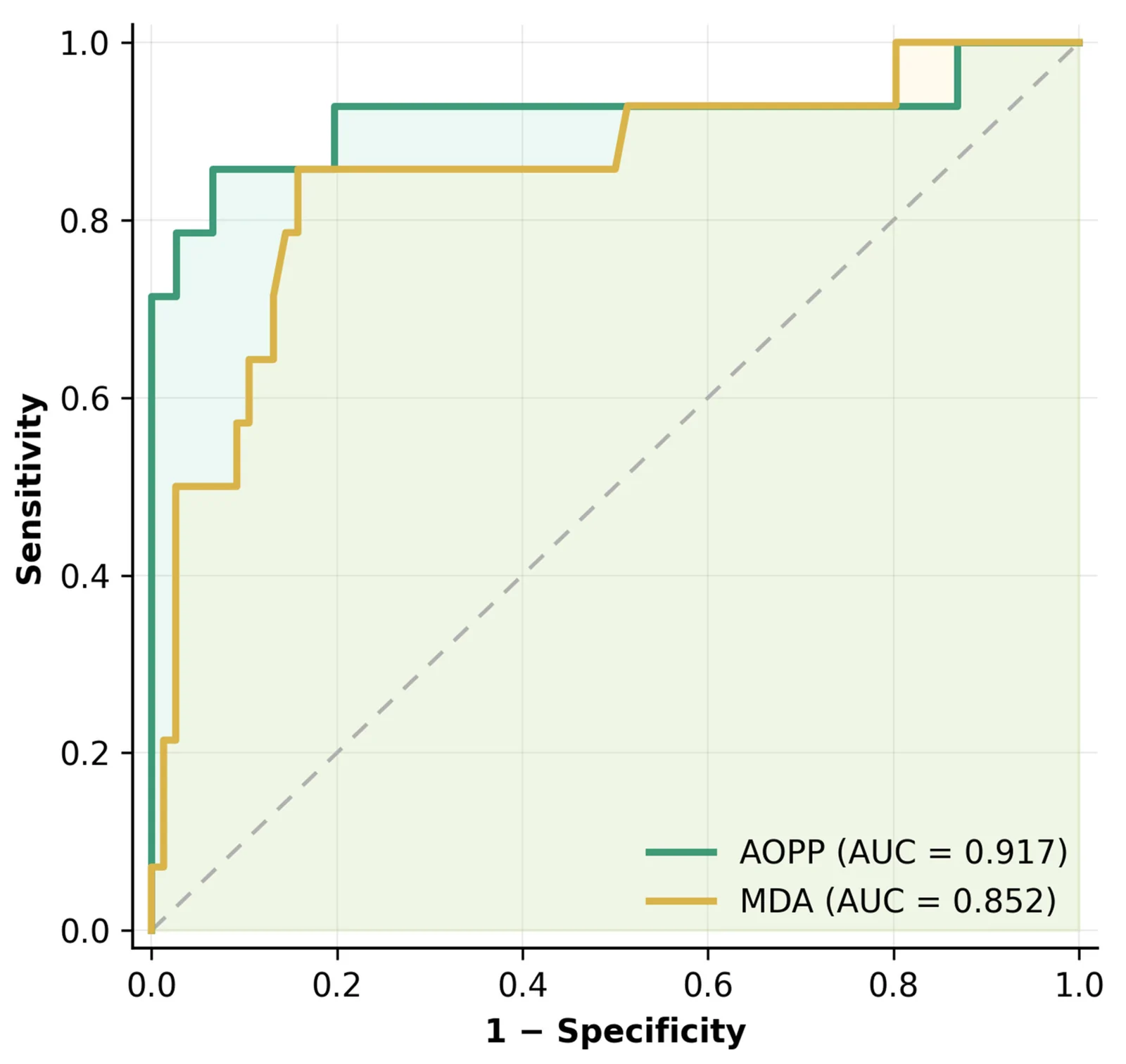
Receiver operating characteristic curves for amniotic fluid AOPP and MDA in relation to NICU admission. Amniotic fluid samples from all participants (*n* = 90) were analyzed; there were 14 NICU admissions. The diagonal dashed line represents the line of no discrimination (area under the curve [AUC] = 0.5); AUC values are shown in the legend. Curves describe apparent, in-sample discrimination; optimism-corrected estimates are given in Table 5. NICU: neonatal intensive care unit.

ROC analysis is summarized in Table 5 and Figure 3. For NICU admission, the apparent area under the curve was 0.917 for AOPP and 0.852 for MDA. Bootstrap optimism correction changed these estimates only marginally, because a single continuous predictor involves no model fitting, but the Youden thresholds themselves were unstable: the bootstrap range for the AOPP threshold spanned 7.28 to 19.58 ng/mL against a point estimate of 14.35 ng/mL. Sensitivity at the selected threshold fell from 0.857 to 0.799 after optimism correction. These thresholds are reported for descriptive purposes and are not proposed for clinical use. Predictive values are reported for NICU admission only, since the 1:1 design fixes prevalence for infection status and would render such values uninterpretable.

The area under the curve for AOPP exceeded that for MDA for NICU admission, but only marginally (difference 0.065, 95% CI 0.003 to 0.127; DeLong z = 2.06, *p* = 0.040). For infection status, the two biomarkers did not differ (difference −0.032, 95% CI −0.139 to 0.076; *p* = 0.563). The comparison should therefore not be read as establishing a general hierarchy between the two markers.

An important caveat applies to the discrimination reported for infection status. Because cases were required to be symptomatic and controls to be asymptomatic, culture-negative and Nugent-negative, the comparison contrasts the two ends of a continuum. This design will tend to widen the observed separation and mechanically inflate the area under the curve reported in Table 5 for infection status.

In a Firth penalized logistic regression model of NICU admission including both biomarkers, AOPP remained associated with admission (odds ratio 1.41 per ng/mL, 95% profile penalized-likelihood CI 1.20 to 1.77, *p* < 0.001) while MDA did not (odds ratio 0.97 per nmol/mL, 95% CI 0.87 to 1.08, *p* = 0.599). The odds ratio for AOPP is expressed per 1 ng/mL; over the interquartile range of AOPP in this cohort, which spans approximately 5 to 12 ng/mL, this corresponds to a substantially larger change in odds.

Because vaginal infection lies upstream of both the biomarkers and the outcome rather than on the causal path between them, infection status was also entered as a candidate covariate. AOPP remained associated with NICU admission in this model (odds ratio 1.54, 95% CI 1.25 to 2.59, *p* = 0.002). The coefficient for infection status could not be estimated with useful precision, spanning a 95% confidence interval from 0.001 to 2.67, as expected with 14 events and three predictors; the point estimate is not interpretable and is therefore not reported. In a model containing infection status alone, the odds ratio for NICU admission was 6.49 (95% CI 1.48 to 41.09, *p* = 0.011).

With 14 events, all regression estimates are unstable and are presented as exploratory. They do not constitute a prediction model, and no threshold, discrimination estimate or odds ratio reported here should be applied clinically without external validation.

## 4. Discussion

In this prospective cohort study of women undergoing elective cesarean delivery, vaginal infection was associated with higher amniotic fluid concentrations of AOPP and MDA, lower cord blood pH and a higher rate of NICU admission compared with uninfected controls. In exploratory analyses, AOPP was associated with NICU admission with an apparent area under the curve of 0.917, and MDA with 0.852. These estimates rest on 14 events, with thresholds derived and evaluated in the same sample, and are therefore hypothesis-generating; they do not establish clinical utility.

A second observation was that the elevation relative to controls was not uniform across infection etiologies. After correction for multiple comparisons, bacterial vaginosis and the symptomatic aerobic bacterial subgroup remained significantly elevated relative to controls for both biomarkers, whereas the mixed, candidiasis and trichomoniasis subgroups did not. This pattern persisted in a sensitivity analysis restricted to the etiologically homogeneous isolated bacterial vaginosis subgroup. It is important, however, to state what this analysis does not show. A Kruskal–Wallis test restricted to the five infection etiologies did not demonstrate a difference between the subgroups themselves. The data therefore do not establish that bacterial etiologies carry greater risk than fungal or protozoal ones. They show only that some subgroups separated from controls and others did not, in a sample with very limited power to detect subgroup differences.

A mechanistic account for these findings can be proposed, although the present study was not designed to test it. AOPP are formed largely through myeloperoxidase-derived chlorinated oxidants acting on plasma proteins [13], and dityrosine-containing AOPP have been reported to act as mediators, rather than only markers, of downstream oxidative and inflammatory signalling [17,18]. Supporting evidence for this mediator role comes from non-obstetric settings, including a rodent model of secondary spinal cord injury in which AOPP activated NADPH oxidase and pro-inflammatory transcriptional pathways [19]; the relevance of these observations to the amniotic compartment is therefore indirect. More broadly, protein and lipid oxidation products such as AOPP and MDA are increasingly recognized as clinically informative biomarkers across a range of inflammatory and metabolic conditions [20]. The vaginal ecosystem has been proposed to be protected by *Lactobacillus*-derived hydrogen peroxide and lactic acid, and loss of *Lactobacillus* dominance is the central pathophysiological event in bacterial vaginosis [21], although the in vivo antimicrobial contribution of hydrogen peroxide itself is disputed [22]. Aerobic vaginitis, as originally defined by a composite wet-mount score [11], is characterized by an overtly increased local inflammatory reaction with elevated interleukin-1, interleukin-6 and interleukin-8 production, in contrast to the comparatively blunted local immune response typical of bacterial vaginosis [3]. Because the aerobic subgroup in the present cohort was not defined by the Donders score, this contrast cannot be applied directly to our data.

An additional consideration is that oxidative stress, by definition, reflects an imbalance between oxidant production and antioxidant defense, yet the present study measured only oxidative damage products, namely AOPP and MDA, without corresponding antioxidant capacity markers such as total antioxidant capacity, superoxide dismutase or glutathione peroxidase [23]. The elevations we observed may therefore reflect increased oxidant generation, reduced antioxidant defense, or both; the available data cannot distinguish these possibilities. Future studies incorporating paired oxidant and antioxidant panels in amniotic fluid would provide a more complete characterization of this redox disturbance.

These findings sit within a heterogeneous clinical literature on vaginal infection and pregnancy outcome. Bacterial vaginosis has been associated with preterm delivery in observational studies [24,25]. Antibiotic treatment, however, has shown inconsistent benefit for preventing preterm delivery despite microbiological cure [26]. This discordance mirrors our own observation that biomarker elevation does not map cleanly onto a single, uniformly treatable pathway. The US Preventive Services Task Force reviewed and ultimately did not recommend universal screening in asymptomatic pregnant women [2]. Aerobic vaginitis has been linked to adverse neonatal outcomes, including NICU admission, in a prospective observational cohort in which the diagnosis was made using a severity-graded aerobic vaginitis score [27], a distinction that should be borne in mind when comparing those findings with ours. For candidiasis, a systematic review and meta-analysis reported no consistent association with adverse perinatal outcomes overall. It nonetheless distinguished symptomatic from asymptomatic infection, with a higher odds ratio in studies in which most participants were symptomatic [28]. Since the candidiasis cases in our cohort were symptomatic, the agreement between our null finding and that meta-analysis is less direct than it may appear. A separate systematic review suggested that treatment of vulvovaginal candidiasis may reduce spontaneous preterm birth [29], although both included trials enrolled women with asymptomatic candidiasis and the pooled estimate rests on a post hoc subgroup analysis of one larger trial, qualifications that the review’s own authors emphasise. Since the candidiasis cases in the present cohort were symptomatic, that evidence is not directly applicable here. Trichomoniasis has been associated with preterm delivery, prelabor rupture of membranes and low birthweight in meta-analytic data [30], and uncertainty persists regarding the effects of treatment in pregnancy [31]. Taken together, this literature does not support a simple hierarchy in which bacterial pathogens carry greater fetal risk than fungal or protozoal ones, and our subgroup findings should not be read as establishing one.

From a translational perspective, amniotic fluid in this study was obtained at the time of cesarean delivery, so AOPP and MDA in their current form provide information at, rather than before, delivery. Any future role would therefore lie in point-of-delivery or postnatal risk stratification rather than in guiding antenatal management, and would depend on a validated assay and on external confirmation that this study cannot provide. Translating these findings into an actionable antenatal test would require a comparable signal in more accessible, non-invasively sampled compartments, such as maternal serum or cervicovaginal fluid, obtained earlier in gestation or at the onset of labor. This would mirror the broader trajectory of biomarker research in intra-amniotic infection toward progressively less invasive sampling [32].

This study has several methodological strengths. The prospective design with an a priori sample size calculation, Nugent scoring for bacterial vaginosis, species-level identification of yeast isolates, culture of Trichomonas vaginalis in a dedicated medium, and exclusion of major oxidative stress confounders strengthen internal validity. The cohort was unusually homogeneous with respect to delivery: all cesareans were elective, membranes were intact in every case, no woman labored, and surgical prophylaxis was uniform, which removes several potential confounders that commonly complicate perinatal biomarker studies. Biomarker measurement was performed by an independent external laboratory on randomly numbered samples, so the analyst could not have known group allocation. Microbiological results were unavailable at delivery, so neither the obstetric nor the neonatal team could have been influenced by them when the admission decision was made. The joint evaluation of two complementary oxidative stress biomarkers, together with etiology-stratified and sensitivity analyses, adds granularity that is largely absent from the existing amniotic fluid biomarker literature.

Several limitations should be acknowledged, and the most consequential concern is measurement. Both assays are commercial immunoassays intended for serum and plasma; neither has been validated for amniotic fluid, and no dilutional linearity or spike-recovery testing was performed. The antibody and calibrator descriptions reported in the Methods derive from technical information supplied by the distributor and could not be verified experimentally, so what the AOPP assay measures cannot be confirmed independently; values in ng/mL are not interchangeable with chloramine-T equivalents obtained by the established spectrophotometric method. Samples were assayed in single wells, precluding study-specific coefficients of variation. Fifteen of 90 MDA measurements (16.7%) fell outside the calibration range, 13 of them below the lower standard and all of these in the control group. This pattern is compatible with a measuring range calibrated for serum, but it is equally compatible with degradation over longer storage, with analytical extrapolation, or with a true biological difference, and the present data cannot distinguish these. Restricting the analysis to in-range values attenuated the MDA difference (r = 0.62 to 0.44) while leaving AOPP essentially unchanged. The MDA findings are therefore semi-quantitative and less secure than those for AOPP.

Second, no direct measurement of intra-amniotic infection or inflammation was made, so ascending microbial spread was not demonstrated and systemic maternal inflammation or common causes of both exposure and outcome remain equally compatible with these data. No participant had leucocytosis, maternal fever or clinically suspected chorioamnionitis, which supports the intended framing of the cohort as women without overt intra-amniotic infection, but this clinical assessment is not a substitute for amniotic culture, cytokine measurement or placental histology. The design also contrasts the two ends of a continuum, since cases were symptomatic and controls asymptomatic and culture-negative, which will widen the observed separation and inflate the discrimination reported for infection status. Two subgroup definitions are imprecise: the aerobic bacterial subgroup was not defined by the Donders score and should not be equated with aerobic vaginitis, and Trichomonas vaginalis was identified by culture rather than by the more sensitive nucleic acid amplification.

Third, admission decisions were made without access to microbiological results, but maternal vaginal symptoms were documented in the clinical record. Awareness of these may have lowered the threshold for admission or for a diagnosis of clinical sepsis, five of which were culture-negative. Results entering the record about three days after delivery may also have informed later management. Storage duration is a further concern, and is a potentially differential pre-analytical variable rather than simply an uncollected one. Because the control arm was filled first and then closed while recruitment for the infection arm continued, and because all samples were assayed in a single batch, controls will on average have been stored longer than cases, by a margin that could approach the full one-week to four-month range. Longer storage would be expected to lower measured concentrations, which is the direction observed in controls, so any such effect would act in favour of the study hypothesis rather than against it. Individual sampling dates were not recorded, and the laboratory holds only the date on which the batch was received rather than per-sample dates, so storage duration could not be reconstructed and its association with the biomarkers could not be examined. Finally, the predictive analyses rest on 14 events with thresholds selected and evaluated in the same sample, so no prediction model is proposed. Group B streptococcus screening is not routine at our institution, C-reactive protein is not measured routinely, and placental pathology and symptom duration were not recorded. Etiology-specific subgroups were small (*n* = 5 to 11), biomarkers were measured at a single time point, and the cohort consisted almost entirely of women undergoing elective repeat cesarean section at a single center, which narrows generalizability.

In summary, amniotic fluid AOPP and MDA were higher in women with vaginal infection at the time of elective cesarean delivery and were associated with lower cord blood pH and more frequent NICU admission. Elevations relative to controls were most pronounced in the bacterial vaginosis and symptomatic aerobic bacterial subgroups, although the etiological subgroups did not differ from one another. Multicenter studies with larger etiology-specific subgroups, assay validation in amniotic fluid against an established reference method, longitudinal sampling across gestation, and concurrent measurement of intra-amniotic inflammation are needed before these patterns can be regarded as established or evaluated for clinical use.

## 5. Conclusions

In this prospective cohort study, vaginal infection at the time of elective cesarean delivery was associated with higher amniotic fluid AOPP and MDA, lower cord blood pH and more frequent NICU admission. Relative to controls, elevations were most pronounced in the bacterial vaginosis and symptomatic aerobic bacterial subgroups, whereas differences could not be demonstrated for candidiasis or trichomoniasis; the etiological subgroups did not differ from one another, and the null results reflect limited power rather than demonstrated absence of effect. Two measurement constraints qualify these findings. Neither assay has been validated for amniotic fluid, and 16.7% of MDA measurements fell outside the manufacturer’s calibration range, so the MDA results are semi-quantitative and should not be read as quantitative estimates of amniotic lipid peroxidation; the AOPP results, although less affected, also require confirmation with an assay validated for this matrix. The associations with neonatal outcome are exploratory, rest on 14 events and were derived and evaluated in the same sample. These findings are hypothesis-generating and support further investigation of amniotic AOPP in larger, externally validated cohorts using an assay validated for this matrix; they do not support clinical use of the biomarkers or thresholds reported here.

## Figures and Tables

**Table 1:** Baseline demographic and clinical characteristics of the study population.

Characteristic	Infection (*n* = 45)	Control (*n* = 45)	*p*-Value	Effect Size
Age, years, median (IQR)	29 (26–33)	26 (24–32)	0.125	r = 0.19
Gravidity, median (IQR)	3 (2–4)	3 (2–4)	0.943	r = 0.01
Parity, median (IQR)	3 (2–3)	3 (2–3)	0.752	r = 0.04
BMI, kg/m^2^, median (IQR)	30.5 (29.0–33.2)	30.2 (28.0–31.9)	0.463	r = 0.09
White blood cell count, ×10^9^/L, median (IQR)	10.0 (7.8–11.4)	9.8 (8.8–11.6)	0.436	r = −0.10
Education, high school or above, *n* (%)	9 (20.0)	10 (22.2)	1.000	
Modern contraceptive method, *n* (%)	20 (44.4)	17 (37.8)	0.669	
Cesarean for previous cesarean, *n* (%)	43 (95.6)	44 (97.8)	1.000	

IQR: interquartile range; BMI: body mass index. Continuous variables were compared with the Mann–Whitney U test and are presented as median (IQR) with the rank-biserial correlation (r) as the effect size; categorical variables were compared with the chi-square or Fisher’s exact test. A positive r indicates a higher mean rank in the infection group. Because r is computed from ranks across the whole distribution, its sign may differ from the direction of the medians when the two distributions overlap substantially, as is the case for the white blood cell count.

**Table 2:** Amniotic fluid AOPP and MDA concentrations by vaginal infection status.

Biomarker	Infection (*n* = 45)	Control (*n* = 45)	*p*-Value	Effect Size (r)
**AOPP, ng/mL,** **median (IQR)**	7.96 (5.84–15.90)	5.77 (3.95–6.71)	<0.001	0.56
**MDA, nmol/mL,** **median (IQR)**	12.49 (8.85–18.06)	7.73 (1.87–10.00)	<0.001	0.62

IQR: interquartile range; r: rank-biserial correlation (Mann–Whitney U test); a positive r indicates a higher value in the infection group. AOPP: advanced oxidation protein products; MDA: malondialdehyde.

**Table 3:** Amniotic fluid AOPP and MDA concentrations stratified by vaginal infection etiology.

Etiology	*n*	AOPP, ng/mL, Median (IQR)	MDA, nmol/mL, Median (IQR)	*p* (AOPP) *	*p* (MDA) *
Control	45	5.77 (3.95–6.71)	7.73 (1.87–10.00)	N/A	N/A
Bacterial vaginosis	10	15.71 (8.25–18.99)	16.47 (11.94–29.23)	0.007 (0.0004)	0.001 (0.0001)
Symptomatic aerobic bacterial growth	10	15.12 (8.15–19.69)	13.84 (11.31–17.64)	<0.001 (<0.0001)	0.005 (0.0003)
BV + candidiasis (mixed)	5	8.91 (7.17–12.42)	12.71 (12.29–17.20)	0.202 (0.014)	0.086 (0.006)
Trichomoniasis	9	6.46 (5.49–7.49)	11.40 (8.85–11.83)	1.000 (0.121)	0.696 (0.046)
Candidiasis	11	6.11 (5.38–7.43)	10.35 (7.67–13.28)	1.000 (0.204)	0.343 (0.023)

* Bonferroni-corrected *p*-value versus the control group for AOPP and MDA respectively (Dunn’s post hoc test following the Kruskal–Wallis test across all six groups); uncorrected *p*-values are given in parentheses. Bonferroni correction was applied to all fifteen pairwise comparisons among the six groups. IQR: interquartile range. The aerobic bacterial subgroup is designated descriptively because the Donders aerobic vaginitis score could not be calculated.

**Table 4:** Perinatal outcomes by vaginal infection status.

Perinatal Outcome	Infection (*n* = 45)	Control (*n* = 45)	*p*-Value	Effect Size
Gestational age at delivery, weeks, median (IQR)	39 (38–39)	39 (39–39)	0.590	r = −0.05
Birth weight, g, mean ± SD	3201 ± 344	3239 ± 488	0.676	d = −0.09
Umbilical cord blood pH, median (IQR)	7.32 (7.30–7.35)	7.37 (7.33–7.38)	0.003	r = −0.36
NICU admission, *n* (%)	12 (26.7)	2 (4.4)	0.007	OR = 7.82 (1.64–37.36)
Apgar score, 1 min, median (IQR)	9 (8–9)	9 (9–9)	0.044	r = −0.20
Apgar score, 5 min, median (IQR)	10 (9–10)	10 (10–10)	0.045	r = −0.21

IQR: interquartile range; SD: standard deviation; NICU: neonatal intensive care unit; OR: odds ratio (95% confidence interval). Cord pH, gestational age and Apgar scores compared with Mann–Whitney U test (effect size = rank-biserial correlation, r); birth weight compared with independent-samples *t*-test (effect size = Cohen’s d); NICU admission compared with Fisher’s exact test. A negative r indicates a lower value in the infection group.

**Table 5:** Receiver operating characteristic analysis and diagnostic performance of amniotic fluid AOPP and MDA for NICU admission and vaginal infection status.

Metric	AOPP → NICU	MDA → NICU	AOPP → Infection	MDA → Infection
Youden threshold	14.35 ng/mL	12.71 nmol/mL	7.96 ng/mL	11.40 nmol/mL
Bootstrap threshold range	7.28–19.58	12.71–26.33	4.87–8.71	6.76–13.33
AUC (95% CI)	0.917 (0.777–0.999)	0.852 (0.708–0.957)	0.778 (0.677–0.867)	0.810 (0.714–0.890)
Optimism-corrected AUC	0.916	0.852	0.778	0.811
Sensitivity (95% CI)	0.857 (0.601–0.960)	0.857 (0.601–0.960)	0.511 (0.370–0.650)	0.644 (0.498–0.768)
Optimism-corrected sensitivity	0.799	0.807	0.491	0.603
Specificity (95% CI)	0.934 (0.855–0.972)	0.842 (0.744–0.907)	1.000 (0.921–1.000)	0.822 (0.687–0.907)
Optimism-corrected specificity	0.928	0.841	0.985	0.786
PPV (95% CI)	0.706 (0.469–0.867)	0.500 (0.314–0.686)	Not applicable	Not applicable
NPV (95% CI)	0.973 (0.905–0.992)	0.970 (0.896–0.992)	Not applicable	Not applicable
Positive likelihood ratio (95% CI)	13.03 (5.44–31.22)	5.43 (3.10–9.52)	Not estimable	3.62 (1.86–7.05)
Negative likelihood ratio (95% CI)	0.15 (0.04–0.55)	0.17 (0.05–0.61)	0.49 (0.36–0.66)	0.43 (0.29–0.66)

AUC: area under the curve; CI: confidence interval; PPV: positive predictive value; NPV: negative predictive value; NICU: neonatal intensive care unit. Thresholds were identified by the Youden index in the same sample in which they were evaluated; the bootstrap threshold range is the 2.5th to 97.5th percentile across 4000 resamples. Optimism-corrected estimates were obtained by bootstrap. Predictive values are not reported for infection status because the 1:1 design fixes prevalence. The positive likelihood ratio for AOPP and infection status is not estimable because specificity was 1.000. The arrow denotes the biomarker evaluated against the stated outcome.

## Data Availability

The data presented in this study are available on request from the corresponding author, subject to institutional ethics approval, due to privacy restrictions on patient-level clinical data.
